# Supplementary material for: Neonatal Screening in Europe Revisited: An ISNS Perspective on the Current State and Developments Since 2010
Source: Int J Neonatal Screen. 2021 Mar 5;7(1):15. doi: 10.3390/ijns7010015 (PMC8006225; doi:10.3390/ijns7010015)
Supplement: Supplementary file 1 [file IJNS-07-00015-s001.zip › Table S1 ISNS Survey 2010-2018.docx]

**ISNS Survey NBS in Europe 2010-2018**

**Table 1**

| **Country:** |  | Remarks |
| --- | --- | --- |
| approx. population 2017 (n) |  |  |
| approx. nr newborns 2017 (n) |  |  |
| number screening labs within programme (n) |  |  |
| number screening labs outside programme (n) |  |  |
| average nr of samples per lab (n) |  |  |
| interval birth-sampling (hours/days) |  |  |
| interval sampling-analysis (days) |  |  |
| type of filterpaper used |  |  |
| percentage coverage of newborns (%) |  |  |
| percentage not screened without reason (%) |  |  |
| information material for parents | yes/no |  |
| informed consent for participation | yes/no |  |
| informed consent for storage of blood spots | yes/no |  |
| length of storage of blood spots (years) |  |  |
| who pays for the screening | government/insurance  /parents/other, i.e. |  |
|  |  |  |

**ISNS Survey NBS in Europe 2010-2018**

**Table 2** (tick the box as appropriate)

| **Condition** | **Within formal**  **NBS system** | **Outside formal**  **NBS system** | **Remarks** |
| --- | --- | --- | --- |
| 3-HMG |  |  |  |
| 3-MCC |  |  |  |
| AA unspecified |  |  |  |
| AC unspecified |  |  |  |
| ARG |  |  |  |
| ASA |  |  |  |
| BIOT |  |  |  |
| BKT/ßKT |  |  |  |
| CAH |  |  |  |
| Cbl A,B |  |  |  |
| CCHD |  |  |  |
| CF |  |  |  |
| CH-T |  |  |  |
| CIT-1 |  |  |  |
| CIT-2 |  |  |  |
| CPT-1 |  |  |  |
| CPT-2 |  |  |  |
| CUD |  |  |  |
| G6PD |  |  |  |
| GA-1 |  |  |  |
| GA-2 |  |  |  |
| GAL |  |  |  |
| Hb S/C |  |  |  |
| Hb S/S |  |  |  |
| Hb S/ß |  |  |  |
| HCSD |  |  |  |
| HCY |  |  |  |
| Hearing |  |  |  |
| HPT-1 |  |  |  |
| IVA |  |  |  |
| LCHADD |  |  |  |
| MADD |  |  |  |
| MCADD |  |  |  |
| MCD |  |  |  |
| MMA |  |  |  |
| MMACBL |  |  |  |
| MSUD |  |  |  |
| MUT |  |  |  |
| PA/PROP |  |  |  |
| PKU |  |  |  |
| SCADD |  |  |  |
| SCHADD |  |  |  |
| SCID |  |  |  |
| TFP |  |  |  |
| TYR-1 |  |  |  |
| TYR-2 |  |  |  |
| UDP |  |  |  |
| VLCADD |  |  |  |
