# Supplementary material for: Neonatal Screening in Europe Revisited: An ISNS Perspective on the Current State and Developments Since 2010
Source: Int J Neonatal Screen. 2021 Mar 5;7(1):15. doi: 10.3390/ijns7010015 (PMC8006225; doi:10.3390/ijns7010015)
Supplement: Supplementary file 1 [file IJNS-07-00015-s001.zip › Table S2 ISNS Survey NBS in Europe 2020.docx]

| **ISNS SURVEY NBS in Europe 2020** |
| --- |
| **Country and author(s) of the report** |
| **Email** |
| **Number of Laboratories in your country dedicated to Newborn Screening (please elaborate if possible)** |
| **Number of newborns screened in 2019 (What is your coverage?)** |
| **Is newborn screening in your country free of charge?** |
| **Numbers and type of disorders you screen for July 1st 2020** |
| **Type of collection paper used** |
| **Is your reporting based on a 5-day week or have you expanded your reporting to weekends for various reasons (if yes, please elaborate)?** |
| **Is oral and / or written informed consent necessary? (If yes, do you think this influence the coverage?)** |
| **What is the recommended sampling time in your country?** |
| **What is the average time between blood sampling and analysis in the screening laboratory?** |
| **How are the Guthrie cards transported from the maternity units to the screening laboratories (regular mail, courier, special conditions)?** |
| **What is the average time until the completion of confirmatory biochemical diagnostic testing? (if it differs depending on the disease please elaborate)** |
| **Do you report normal results to the parents? If yes, by which method? (eg mail, online etc)** |
| **Do you take repeat DBS samples? For which disorders, when and how?** |
| **How do you report abnormal results from the screening laboratory and do you ask for a new DBS sample together with the diagnostic testing?** |
| **Is there available information material for prospective parents? If yes, in what form (eg online, pamphlet)?** |
| **Is there a specific policy regarding storing of Guthrie cards? (storing time?)** |
| **Do you use internal/external Quality Controls? If yes, which one(s)?** |
| **Did you observe a positive/negative trend compared to previous years as far as the numbers of newborns screened?** |
| **Did you observe a positive/negative trend compared to previous years as far as disease frequency is concerned for any of the screened disorders?** |
| **Please elaborate on any changes in Newborn screening in your country as far as the following is concerned: 1. Extra disorders/tests added or removed 2. Important changes in cutoff limits or ways for deciding recalls? 3. Prominent analytical developments? 4. New pilot studies (how would you provide external quality assurance to these tests)? 5. Numbers of screening laboratories in your country? 6. Changes in equipment? 7. Newborn screening policies? 8. Anything unusual during the past year (eg. surviving an earthquake)** |
| **Please elaborate on any plans for the future you might have** |
| **Publications in peer-reviewed journals, annual epidemiological reports** |
| **Anything further important that has happened in your country the last year that you wish to add?** |
|  |
